# Assessment of the Antitumor Potentiality of Newly Designed Steroid Derivatives: Pre-Clinical Study

**DOI:** 10.31557/APJCP.2019.20.10.3057

**Published:** 2019

**Authors:** Dina S El-Kady, Naglaa A Ali, Alaa H Sayed, Mervat M Abdelhalim, Gamal A Elmegeed, Hanaa H Ahmed

**Affiliations:** 1 *Department of Hormones, Medical Research Division, National Research Centre, Dokki, Giza, Egypt,*; 2 *Department of Applied Medical Sciences, Community College in Al-Qurayyat, Al-Jouf University, KSA.*

**Keywords:** Hepatocellular carcinoma, heterosteroids, tumor markers, angiogenesis, inflammation

## Abstract

Cancer is recognized as one of the most prevalent contributors to mortality in several nations and it remains one of the common health issues globally. In particular, hepatocellular carcinoma (HCC) has become a public health problem along with the increase of hepatitis B (HBV) and hepatitis C (HCV) virus infections. Based on this fact, our study goaled to synthesize newly hybrid drugs containing heterocyclic rings incorporated to steroid moiety and to examine the potential antitumor activity of the newly designed heterosteroid derivatives against HCC induced in animal model. Several heterocyclic steroids were synthesized** 2-7** and confirmed *via* the analytical and spectral data (IR, ^1^H NMR^13^C NMR and Mass spectroscopy). Compounds **3, 4**, and **5** were chosen to be investigated as anticancer agents in HCC rat model by means of validated biomarkers (alfa –fetoprotein, endoglin, lipocali-2 and heat shock protein-70). Following administration of compounds **3, 4** or **5**, availability of the active tumor marker molecules was significantly dropped and a substantial decrease of the angiogenic and inflammatory mediators was also evident. These findings were supported by the histological examination of liver tissue. Taken together, this study indicates the potential anticancer activity of the newly synthesized heterosteroid derivatives against HCC *in vivo*. The antitumor activity of these compounds was likely attributable to modulating some signal transduction pathways involved in tumorigenesis, angiogenesis and inflammation.

## Introduction

Hybrid anticancer drugs are considered as new therapeutic approach for cancer as they have a potency to overcome most of the pharmacokinetic drawbacks encountered with the use of traditional anticancer medications (Rahman et al., 2007). Therefore, these drugs are gaining approval as a therapeutic option for different forms of cancer (Tantawy et al., 2017).

Steroids have been a prime target of studies not only due to their fascinating structural framework, but also owing to their astonishing array of biological activities. Steroids have a powerful ability to pass through the cell membranes and attach to the membrane and nuclear receptors. Additionally, even a small change in the steroid molecule can evoke strong pharmacological effects (Brown et al., 1985). Mohamed et al., (2005) proved that several steroid derivatives, bearing heterocyclic systems as a part of rings-A and -D; display various pharmacological properties, like anti-microbial, anti-inflammatory, hypotensive, hypocholesterolemic and diuretic activities.

Backbone of literature revealed that the incorporation of hetero atom (N/O/S) with the steroid molecules increases the biological potency of these molecules (Gupta et al., 1996) particularly nitrogen containing steroids which showed a potent ability to monitor a variety of biological cascades. Therefore, they represent potential drug candidates for the treatment of a great number of diseases including breast cancer (Visvanathan, and Davidson, 2003), prostate cancer (Li et al., 1995), hepatocellular carcinoma (He et al., 1999; El-Far et al., 2009; Mohamed et al., 2010), autoimmune diseases (Latham et al., 2003) and osteoporosis (Hosking et al., 1998).

Cancer is described as one of the most prevalent contributors to mortality in several nations (Varmus, 2006). Despite the huge efforts to develop novel chemotherapeutics for the treatment of various types of cancer, this ailment remains one of the common health problems throughout the globe. Hepatocellular carcinoma (HCC) constitutes the 5th cancer type in men and the 7th in women, with more than 748,000 new cases being diagnosed annually, representing 9.2% of all new cancer cases all over the world (Jemal et al., 2011). The number of new cases of HCC upregulates year after year and 84%, of all HCC patients globally lives in resource-poor countries (Kew, 2014). The incidence of HCC varies significantly around the world, indicating the prevalence of its major causative factors like hepatitis B (HBV) and hepatitis C (HCV) virus infections, heavy alcohol consumption and great dietary impact to aflatoxins (Kirk et al., 2004).

In Egypt, HCC is the 2^nd^ common tumor in men and the 5th in women. The main risk factor is HCV infection, which is responsible for 20% of acute hepatitis, 70% of chronic hepatitis, 40% of cirrhosis and up to 90% of HCC cases (Hosny et al., 2004). The increasing incidence of HCC and the limited therapeutic opportunities opened up a line of thought about the urgent needs for exploration of substitutional therapy for this type of tumor.

Therefore, the main target of this research work was to design and synthesize new hybrid compounds containing steroids and heterocyclic rings to be examined as antitumor molecules against hepatocellular carcinoma in the experimental model. 

## Materials and Methods

*Chemistry*

Starting steroids molecules were purchased from Sigma Company, USA. All solvents were anhydrated by distillation prior to using. All melting points were measured using an electrothermal apparatus. The IR spectra were recorded in (KBr discs) using Shimadzu FT-IR 8201 PC spectrometer and expressed in cm^-1^. The ^1^H NMR and ^13^C NMR spectra were registered by using Jeol instrument (Japan), at 270 and 125 MHz respectively, in DMSO-d_6_ as solvent and the chemical shifts were recorded in ppm relative to TMS. The spin multiplicities were abbreviated by the letters: s-singlet, d-doublet, t-triplet, q-quartet and m –multiplet (more than quartet). Mass spectra were recorded on a GCMS-QP 1000 ex spectra mass spectrometer operating at 70 ev. Elemental analyses were carried out by the Microanalytical Data Unit at the National Research Centre, Giza, Egypt and the Microanalytical Data Unit at Cairo University, Giza, Egypt. The reactions were monitored by thin layer chromatography (TLC) which was carried out using Merck 60 F254 aluminum sheets and visualized by UV light (254 nm). The mixtures were separated by preparative TLC and gravity chromatography. All steroid derivatives showed the characteristic spectral data of cyclopentanoperhydrophenanthrene nuclei of androstane series that were similar to those reported in the literature (Fuente et al., 2005).

*Synthesis of compound (* ***2*** *)*

A mixture of acetylated testosterone **1** (0.33g, 1mmol), 4-methoxybenzaldehyde (0.13 g, 1mmol) and malononitrile (0.06 g, 1mmol) in absolute ethanol (10 mL) containing ammonium acetate (0.98 g, 2% excess) was heated under reflux for 4-5 hours until all starting materials disappeared as indicated by TLC. The reaction mixture was poured into ice/water mixture and neutralized with dilute HCl. The formed solid product was collected by filtration, dried and crystallized from absolute ethanol to afford compound **2.**

(1S,3aS,3bR,11aR,11bS,13aS)-8-amino-9-cyano-10-(4-methoxyphenyl)-11a,13a-dimethyl-2,3,3a,3b,4,5,6a,7,11,11a,11b,12,13,13a-tetradecahydro-1H-cyclopenta-5,6-naphtho-1,2-g-quinolin-1-yl acetate (**2**).

Pale brown powder, from absolute ethanol: Yield = (0.70 g) 65 %, mp 55-57 ^o^ C. IR (kBr, cm^-1^): ν 3500-3400 (NH_2_, NH), 2935, 2854 (CH-aliphatic), 2220 (CN), 1725 (Ac, C=O), 1606 (C=C). ^1^H NMR (DMSO-d_6_, ppm): δ = 0.78 (s, 3H, CH_3_-19), 1.20-2.27 (m, steroid moiety), 1.23 (s, 3H, CH_3_-18), 2.25 (s, 3H, CH_3_-Ac), 3.83 (d, 1H, C_3_-H), 3.88 (s, 3H, OCH_3_), 5.61 (d, 1H, C_4_-H), 7.05-7.19 (m, 4H, aromatic-H), 8.00 (d, 2H, NH_2_, D_2_O-exchangeable), 8.50 (s, 1H, NH, D_2_O-exchangeable). ^13^C NMR (DMSO-d_6_, ppm): δ = 33.6 (C-1), 132.2 (C-2), 52.8 (C-3), 110.2 (C-4), 165.4 (C-5), 33.1 (C-6), 31.9 (C-7), 35.5 (C-8), 54.7 (C-9), 40.0 (C-10), 54.7 (C-11), 23.5 (C-12), 38.9 (C-13), 42.6 (C-14), 23.7 (C-15), 27.6 (C-16), 82.7 (C-17), 12.2 (C-18), 23.6 (C-19), 170.2 (C=O), 21.0 (C-CH_3_), 116.6, 141.3 (C=C), 115.8 (C-CN), 132.1, 133.6 (C=C),172.5 (C-NH), 114.2, 124.8, 130.0, 159.8 (C-aromatic), 55.8 (C-OCH_3_). MS (EI) m/z =512 M+˙, 46%, 496 (26), 486 (35), 481 (25), 439 (16), 422 (98). Calc M.wt for C_32_H_39_N_3_O_3_ (512.30).

*General procedure for synthesis of compounds (* ***3*** *) and (* ***4*** *)*

To a solution of compound **2** (0.57 g, 1mmol) in 15 ml potassium ethoxide (10%), carbon disulfide (1 mmol, 0.07 g) was added for the synthesis of compound **4** and excess of carbon disulfide was added (0.21 g) for the synthesis of compound **3**. The reaction mixture was heated under reflux for 4 hours until all starting materials disappeared as indicated by TLC. The solvent was evaporated under vacuum and the remaining oil was treated with ice/water mixture and neutralized with dilute HCL. The obtained solid product was collected by filtration, dried, and crystallized from absolute ethanol.

(1S,3aS,3bR,13aR,13bS,15aS)-12-(4-methoxyphenyl)-13a,15a-dimethyl-9,11-dithioxo-2,3,3a,3b,4,5,6a,7,8,9,10,11,13,13a,13b,14,15,15a-octadecahydro-1H-cyclopenta-5,6-naphtho-1,2-g-pyrimido-4,5-bquinolin-1-yl acetate (**3**)

Brown powder, from absolute ethanol: Yield= (0.50 g) 87 %, mp 115-118^o^C. IR (kBr, cm^-1^): ν 3434-3385 (3NH), 2937, 2848 (CH-aliphatic), 1728 (Ac, C=O), 1617 (C=C), 1193, 1175 (2C=S). ^1^H NMR (DMSO-d_6_, ppm): δ = 0.96 (s, 3H, CH_3_-19), 1.20 (s, 3H, CH_3_-18), 1.20-2.57 (m, steroid moiety), 2.22 (s, 3H, CH_3_-Ac), 3.85 (d, 1H, C3-H), 3.89 (s, 3H, OCH_3_), 4.45 (d, 1H, C4-H), 7.06-7.19 (m, 4H, aromatic-H), 7.89 (s, 1H, 1NH, D_2_O-exchangeable), 9.94 (s, 2H, 2NH, D_2_O-exchangeable). ^13^C NMR (DMSO-d_6_, ppm): δ = 35.7 (C-1), 130.2 (C-2), 52.4 (C-3), 111.5 (C-4), 166.3 (C-5), 34.1 (C-6), 31.9 (C-7), 35.5 (C-8), 54.7 (C-9), 40.0 (C-10), 54.7 (C-11), 23.5 (C-12), 38.9 (C-13), 42.6 (C-14), 23.7 (C-15), 27.6 (C-16), 82.7 (C-17), 12.2 (C-18), 23.6 (C-19), 170.2 (C=O), 21.0 (C-CH_3_), 116.6, 141.3 (C=C), 132.1, 133.6 (C=C), 172.5 (C-NH), 114.2, 124.8, 130.0, 159.8 (C-aromatic), 55.8 (C-OCH_3_), 178.2 (C=S), 194.9 (C=S). MS (EI) m/z = 589 M+˙, 35%, 555 (10), 526 (18), 496 (16), 479 (57), 420 (56). Calc M.wt for: C_33_H_39_N_3_O_3_S_2_ (589.243).

(1S,3aS,3bR,13aR,13bS,15aS)-12-(4-methoxyphenyl)-13a,15a-dimethyl-11-oxo-9-thioxo-1,2,3,3a,3b,4,5,6a,7,8,9,11,13,13a,13b,14,15,15a-octadecahydrocyclopenta-5,6-naphtho-1,2-g-1,3-thiazino-4,5-bquinolin-1-yl acetate (**4**)

Pale yellow powder, from absolute ethanol: Yield = (0.47g) 81 %, mp=130-134 ^o^ C. IR (kBr, cm^-1^): ν 3456-3380 (2NH), 2937, 2845 (CH-aliphatic), 1730 (Ac, C=O), 1685 (C=O), 1509 (C=C), 1185 (C=S). ^1^H NMR (DMSO-d_6_, ppm): δ = 0.92 (s, 3H, CH_3_-19), 1.20 (s, 3H, CH_3_-18), 1.22-2.50 (m, steroid moiety), 2.48 (s, 3H, CH_3_-Ac), 3.41 (d, 1H, C3-H), 3.86 (s, 3H, OCH_3_), 5.44 (s, 1H, C4-H), 6.78-7.32 (m, 4H, aromatic), 8.00 (s, 2H, 2NH, D_2_O-exchangeable). ^13^C NMR (DMSO-d_6_, ppm): δ = 35.7 (C-1), 132.1 (C-2), 54.9 (C-3), 110.5 (C-4), 166.2 (C-5), 33.1 (C-6), 31.9 (C-7), 35.5 (C-8), 54.7 (C-9), 40.0 (C-10), 54.7 (C-11), 23.5 (C-12), 38.9 (C-13), 42.6 (C-14), 23.7 (C-15), 27.6 (C-16), 82.7 (C-17), 12.2 (C-18), 23.6 (C-19), 170.2, 172.0 (2C=O), 21.0 (C-CH_3_), 116.6, 141.3 (C=C), 132.1, 133.6 (C=C), 172.5 (C-NH), 114.2, 124.8, 130.0, 159.8 (C-aromatic), 55.8 (C-OCH_3_), 178.2 (C=S), 163.7 (C=NH), 194.7 (C-S). MS (EI) m/z = 589 M+˙-1, 52%, 559 (21), 547 (91), 527 (20), 483 (30), 302 (46). Calc M.wt for C_33_H_38_N_2_O_4_S_2_ (590.227).

*General procedure for the synthesis of compounds (* ***5*** *) and (* ***6*** *)*

To a solution of compound **2** (1mmol, 0.51g) in absolute ethanol (30 mL) containing a catalytic amount of triethylamine (0.5 mL), equimolar amount of hydrazines namely, phenyl hydrazine (1 mmol, 0.10 g) or hydrazine hydrate (1 mmol, 0.05 g) was added. The reaction mixture, in each case, was heated under reflux for 3-5 hours until all the starting material disappeared as indicated by TLC. The reaction mixture was poured into ice/water mixture and neutralized with dilute HCl. The formed solid product in each case was collected by filtration, dried and crystallized from appropriate solvent.

(1S,3aS,3bR,12aR,12bS,14aS)-10-imino-11-(4-methoxyphenyl)-12a,14a-dimethyl-9-phenyl-1,2,3,3a,3b,4,5,6a,7,8,9,10,12,12a,12b,13,14,14a-octadecahydrocyclopenta-5,6-naphtho-1,2-g-pyrazolo-3,4-b-quinolin-1-yl acetate (**5**)

Brown powder from absolute ethanol: Yield (0.11g) 46 %, mp = 99-102 ^o^C. IR (kBr, cm^-1^): ν 3433-3300 (3NH), 2943, 2845 (CH-aliphatic), 1728 (Ac, C=O), 1662 (C=N), 1606 (C=C). ^1^H NMR (DMSO-d_6_, ppm): δ = 0.90 (s, 3H, CH_3_-19), 1.18 (s, 3H, CH_3_-18), 1.23-2.51 (m, steroid moiety), 2.00 (s, 3H, CH_3_-Ac), 3.76 (d, 1H, C3-H), 3.82 (d, 3H, OCH_3_), 5.51 (s, 1H, C4-H), 6.88-7.56 (m, 9H, aromatic-H), 9.00 (s, 1H, NH, D_2_O-exchangeable), 10.10 (s, 2H, 2NH, D_2_O-exchangeable). ^13^C NMR (DMSO-d_6_, ppm): δ = 34.0 (C-1), 132.6 (C-2), 53.3 (C-3), 110.3 (C-4), 165.7 (C-5), 33.0 (C-6), 31.9 (C-7), 35.5 (C-8), 54.2 (C-9), 44.0 (C-10), 33.5 (C-11), 36.9 (C-12), 42.8 (C-13), 50.7 (C-14), 23.5 (C-15), 27.6 (C-16), 82.7 (C-17), 12.2 (C-18), 23.6 (C-19), 133.6, 132.1 (C=C), 170.2 (C=O), 55.8 (C-OCH_3_), 150.1 (C=NH), 114.2, 130.1, 127.0 (aromatic C), 122.8, 123.9, 136.2 (aromatic C). MS (EI) m/z = 604 M+˙, 25%, 573 (73), 545 (43), 527 (12), 497 (40), 484 (43). Calc M.wt for C_38_H_44_N_4_O_3_ (604.341).

(1S,3aS,3bR,12aR,12bS,14aS)-10-imino-11-(4-methoxyphenyl)-12a,14a-dimethyl-1,2,3,3a,3b,4,5,6a,7,8,9,10,12,12a,12b,13,14,14a-octadecahydrocyclopenta5, 6naphtho1,2-gpyrazolo-3,4-b-quinolin-1-yl acetate (**6**)

Pale brown from absolute ethanol: Yield = (0.20 g) 97 %, mp: 122-124 oC. IR (kBr, cm^-1^): ν 3437-3290 (4NH), 2943, 2847 (CH-aliphatic), 1729 (Ac, C=O), 1658 (C=N), 1621 (C=C). ^1^H NMR (DMSO-d_6_, ppm): δ =0.90 (s, 3H, CH_3_-19), 1.60-2.32 (m, steroid moiety), 1.97 (s, 3H, CH_3_-18), 2.49 (s, 3H, CH_3_-Ac), 3.46 (d, 1H, C3-H), 3.82 (s, 3H, OCH_3_), 5.42 (s, 1H, C4-H), 6.81-7.02 (m, 4H, aromatic-H); ^13^C NMR (DMSO-d_6_, ppm): δ =34.0 (C-1), 130.9 (C-2), 53.7 (C-3), 111.0 (C-4), 166.2 (C-5), 33.5 (C-6), 31.9 (C-7), 35.5 (C-8), 54.2 (C-9), 44.0 (C-10), 33.5 (C-11), 36.9 (C-12), 42.8 (C-13), 50.7 (C-14), 23.5 (C-15), 27.6 (C-16), 82.7 (C-17), 12.2 (C-18), 23.6 (C-19), 133.6, 132.1 (C=C), 170.2 (C=O), 55.8 (C-OCH_3_), 150.1 (C=NH), 114.2, 130.1, 127.0 (aromatic C). MS (EI) m/z = 528 M+˙, 27%, 497 (68), 484 (18), 421 (84), 408 (74), 362 (37). Calc M.wt. for C_32_H_40_N_4_O_3_ (528.310).

*Synthesis of compound (* ***7*** *)*

To a mixture of compound **2** (0.51g, 1mmol) and guanidine hydrochloride (0.09 g, 1mmol) in 30 mL absolute ethanol, a catalytic amount of triethylamine was added (0.5 mL). The reaction mixture was heated under reflux for 4 hours until all the starting materials disappeared as indicated by TLC. The reaction mixture was left to cold at room temperature and poured over ice/water mixture. The formed solid product was collected by filtration dried and crystallized from absolute ethanol to form compound **7**.

(1S,3aS,3bR,13aR,13bS,15aS)-9,11-diimino-12-(4-methoxyphenyl)-13a,15a-dimethyl-2,3,3a,3b,4,5,6a,7,8,9,10,11,13,13a,13b,14,15,15a-octadecahydro-1H-cyclopenta-5,6-naphtho-1,2-g-pyrimido-4,5-b-quinolin-1-yl acetate (**7**)

Gray powder from absolute ethanol: Yield (0.40g) 92 %; mp = 119-122 ^o^C; IR (KBr cm^-1^ ): ν 3435-3292 (5NH), 2943, 2849 (CH-aliphatic), 1730 (Ac, C=O), 1655 (C=N), 1620 (C=C).^1^H NMR (DMSO-d_6_, ppm): δ = 0.76 (s, 3H, CH_3_-19), 1,16 (s, 3H, CH3-18), 1.61-2.53 (m, steroid moiety), 3.46 (d, 1H, C3-H), 3.89 (s, 3H, OCH_3_), 5.50 (d, 1H, C4-H), 6.11 (s, 1H, NH, D_2_O-exchangeable), 6.88-7.59 (m, 9H , aromatic-H, 4NH). ^13^C NMR (DMSO-d_6_, ppm): δ =33.8 (C-1), 131.2 (C-2), 53.5 (C-3), 110.2 (C-4), 166.7 (C-5), 33.5 (C-6), 32.9 (C-7), 35.5 (C-8), 54.3 (C-9), 45.0 (C-10), 33.5 (C-11), 37.2 (C-12), 42.8 (C-13), 50.2 (C-14), 23.5 (C-15), 27.6 (C-16), 82.0 (C-17), 12.7 (C-18), 23.2 (C-19), 133.6, 132.1 (C=C), 170.2 (C=O), 55.8 (C-OCH_3_), 148.3, 150.4 (2C=NH), 114.2, 130.1, 127.0 (aromatic C). MS (EI) m/z = 556 M+˙+1, 34%, 524 (30), 529 (22), 448 (39), 422 (28), 363 (21). Calc M.wt. for C_33_H_41_N_5_O_3_ (555.321).

*Biological assay*

*Animals*

Sixty adult female albino rats of Wistar strain weighing 180-190 g were acquired from a breeding stock colony maintained in the Animal House of the National Research Centre, Giza, Egypt. Animals were housed in stainless steel wire meshed cages under environmentally controlled conditions with respect to light, temperature or air humidity and fed with standard laboratory food and water ad libitum. All protocols and procedures were approved by Institutional Ethics Committee of the National Research Centre, Giza, Egypt and the experiments were performed as per guideline of National Research Centre Ethical Committee for Medical Research.

*Experimental setting*

After an acclimatization period of 10 days, rats were randomly distributed into 6 groups (10 rats /group). Gp (1): Negative control group, which orally received vehicle solution (DMSO;10 % in 0.9 % normal saline) 1mL/rat, five times a week during the whole experimental period (12 weeks), Gp (2): HCC group, which was orally administered N-nitrosodiethylamine (NDEA)] Sigma-Aldrich Chemical Company, St. Louis, MO,USA[ in a dose of 20 mg/kg b.wt., five times a week for 6 weeks, according to the modified method of Darwish and El-Boghdady (2009), Gp (3): Doxorubicin-treated group in which the HCC bearing rats, were intraperitoneally (i.p.) treated with doxorubicin (DOXO)] Pharmacia Italia S.P.A, Milan, Italy[ as a reference drug in a dose of 0.72 mg/rat (equivalent to the human dose of 40 mg/m^2^) according to Barnes and Paget (1965) once a week for 6 weeks, Gp (4): Compound **5**- treated group, in which the HCC bearing rats, were i.p. treated with compound **5 **(dissolved in DMSO) in a daily dose of 10 mg/kg b.wt. for two weeks followed by treatment with the same dose (2 times/ week) for four weeks according to the modified method of Sreepriya and Bali (2005), Gp (5): Compound **4**-treated group in which the HCC bearing rats , were i.p. treated with compound **4** (dissolved in DMSO) in a daily dose of 10 mg/kg b.wt. for two weeks followed by treatment with the same dose (2times/week) for four weeks and Gp (6): Compound **3**-treated group, in which the HCC bearing rats , were i.p. treated with compound **3** (dissolved in DMSO) in a daily dose of 10 mg/kg b.wt. for two weeks followed by treatment with the same dose (2times/week) for four weeks. 

After the completion of this round (12 weeks), rats were fasted overnight and blood samples were withdrawn, under diethyl ether anesthesia, from the retro-orbital venous plexus in centrifuge tubes free from anticoagulant to separate serum samples for biochemical analyses. After blood collection, the rats were sacrificed by cervical dislocation, and a midline abdominal incision was performed and liver was quickly dissected out and washed in ice cold saline and then preserved in 10 % neutral formalin solution for histopathological examination.

*Biochemical determinations*

Serum ALP, AST, ALT activity was determined by spectrophotometric method using kits purchased from Chrono Lab, Barcelona, (Spain) according to the methods described by Young et al., (2001). Urea and creatinine serum levels were estimated by spectrophotometric method using kits obtained from Chrono lab Barcelona, (Spain) following the methods of Wenger et al., (1984).

Serum levels of alpha-fetoprotein (AFP) and endoglin (ENG) were estimated using rat ELISA kits purchased from Elabscience Biotechnology Co., Ltd (P.R.C.), according to the manufacturer’s instructions provided with the assay kits. Serum levels of lipocalin-2 (LCN-2) and heat shock protein-70 (HSP-70) were quantified using rat ELISA kits obtained from WKEA MED Supplies Corp Co., Ltd (China), according to the manual instructions provided with the assay kits. 

*Histophathological procedure*

The fixed liver specimens were trimmed, washed and dehydrated in ascending grades of alcohol. Tissue specimens were then cleared in xylene, embedded in paraffin, sectioned at 4-6 microns thickness, stained with Hematoxylen and Eosin (H and E stain) and examined under the light microscope (Carleton, 1976). 

*Statistics*

In the present study, all results were expressed as Mean+S.E of the mean. Data were analyzed by one-way analysis of variance (ANOVA) using the Statistical Package for the Social Sciences (SPSS) program, version 21, followed by least significant difference (LSD) to compare significance between groups (Armitage and Berry, 1987). Difference was considered significant when P value was < 0.05.

## Results

*Chemistry*

**Scheme 1:**
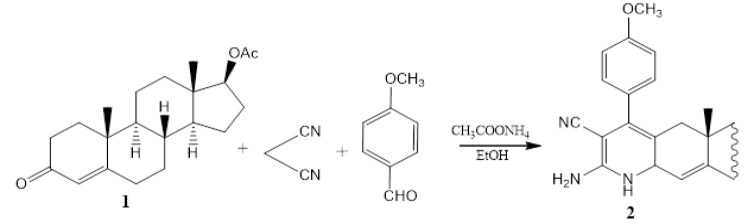
Multi-Component Reaction for the Synthesis of Compound 2

**Scheme 2:**
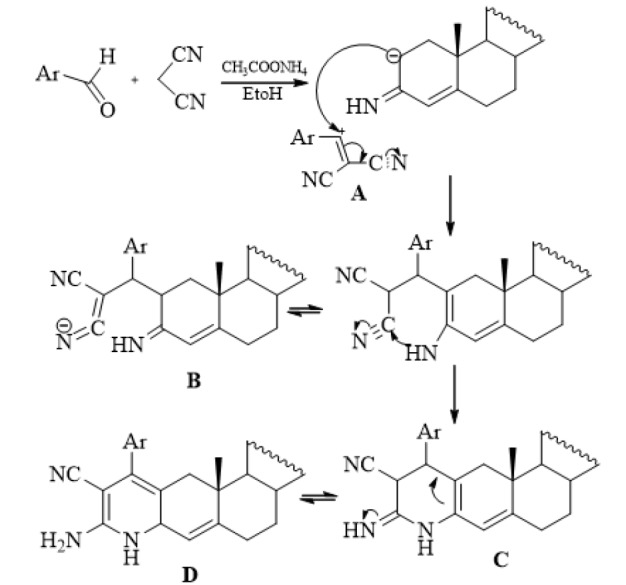
Mechanism for the Synthesis of Compound 2

One pot multi-component reactions (MCRs) were attempted as a straight forward method for the synthesis of heterocyclic steroids. MCRs are one of the best tools in combinatorial chemistry owing to their productivity, simple procedures, convergence, and facile execution (Terrett, 1998). Reaction of acetylated testosterone **1 **with equimolar amounts of p-methoxybenzaldhyde and malononitrile in absolute ethanol containing a catalytic amount of ammonium acetate were performed to afford the corresponding aminopyridoandrostene derivative **2** (Scheme 1). The formation of compound **2** can be explained by the probable mechanism represented in Scheme (2). The reaction occurs through initial production of the intermediate acrylonitrile A followed by its nucleophilic attack of the anion of testosterone to produce the intermediate B. The final product D was produced through the initial cyclization and subsequent tautomerization of the cyclic intermediate C. The IR spectrum of compound **2** shows the presence of CN and NH_2_ groups stretching at 2,220 and 3,429 cm^-1 ^respectively. The ^1^HNMR spectrum reveals characteristic signals of steroid moiety, in addition to the presence of the characterized signals for OCH_3_, NH_2_, NH, C4-H, C3-H, Ac-CH_3_ (c.f. Materials and Methods).

**Scheme 3:**
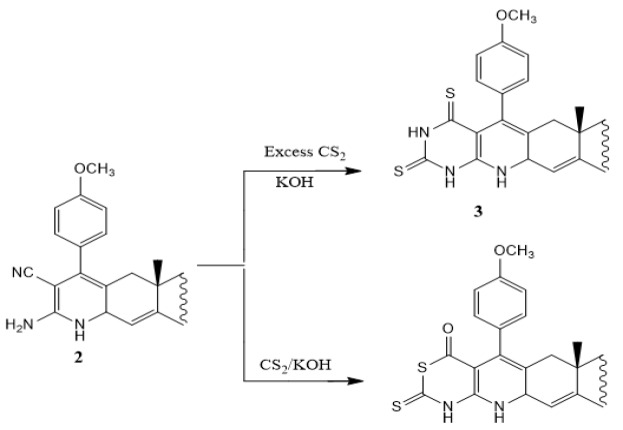
Synthesis of Compounds 3 and 4

**Scheme 4:**
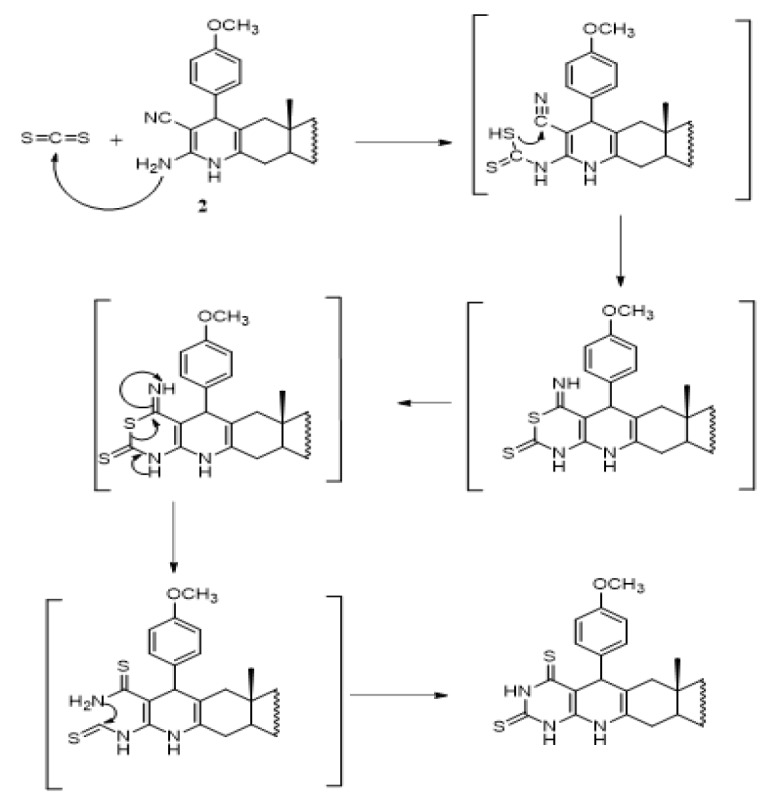
Mechanism for the Synthesis of Compounds 3 and 4

Compound **2** reacts with equimolar amount of carbon disulfide in alcoholic potassium hydroxide solution (10%) to form thioxothiazinopyridoandrostene derivative **4**. On the other hand, exceeding the amount of carbon disulfide affords dithioxopyrimidinopyrido-androstene derivative **3** (Scheme 3). The formation of compounds **3** and **4** can be explained by the possible mechanism represented in Scheme (4) (Smolyar and Yutilov, 2009). The IR spectrum of compounds **3** and **4** exhibit strong sharp absorption band at 3,380 cm^-1^ attributable to NH groups and devoid any band for CN. IR spectrum of compound **3** shows sharp absorption bands at 1193 and 1175 for the two C=S groups. IR spectrum of compound **4** shows sharp absorption band at 1185 for one C=S group, and two absorption bands at 1,730 (Ac, C=O) and 1,685 (oxothiazine, C=O). ^1^H NMR spectra of compounds **3** and **4** exhibit, in addition to the expected signals of steroid moiety, the characterized signal of NH groups and OCH_3_ group. Also, EIMS agrees well with the proposed structure (c.f. Material and Methods).

**Scheme 5:**
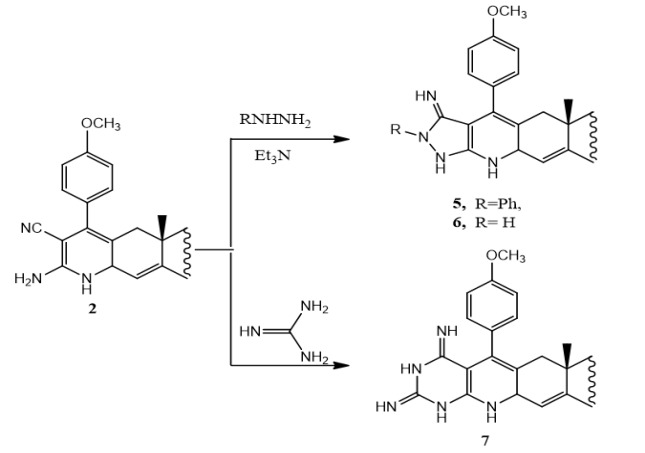
Synthesis of Compounds 5,6 and 7

**Scheme 6:**
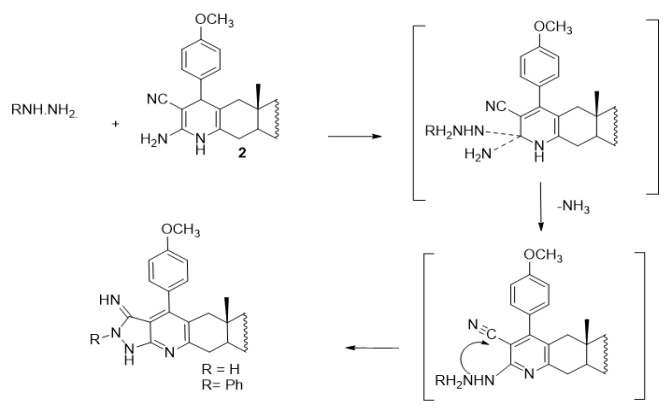
Mechanism for the Synthesis of Compounds 5 and 6

**Scheme 7:**
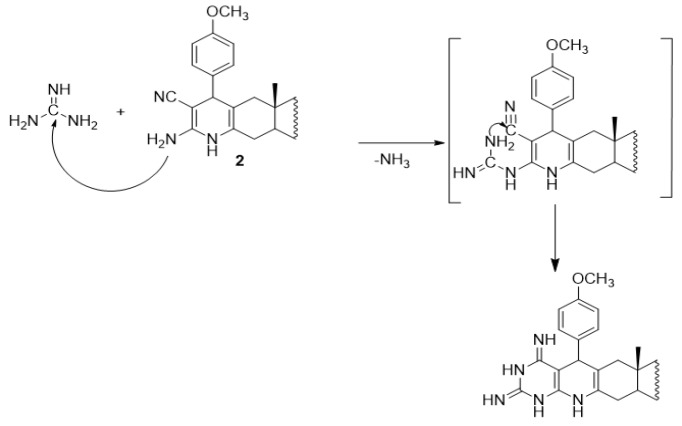
Mechanism for the Synthesis of Compound 7

Furthermore, treatment of compound **2** with equimolar amount of hydrazines namely, hydrazine hydrate or phenyl hydrazine in absolute ethanol containing a catalytic amount of triethylamine yields the pyrazolopyridoandrostene derivatives **5** and **6** respectively (Scheme 5). The formation of compounds **5** and **6** takes place *via* the possible mechanism represented in Scheme (6) (Chubb and Edward, 1981). Also, when compound **2 **is allowed to react with guanidine hydrochloride in boiling ethanol in the presence of triethylamine as a catalyst, afforded the primidinopyridoandrostene derivative **7** (Scheme 5). The formation of compound **7** can be explained by the possible mechanism represented in Scheme (7). IR, ^1^H NMR, ^13^C NMR and EIMS data of compounds **5, 6 **and **7 **are consistent with the proposed structures (c.f. Material and Methods).

## Discussion

*Biological assay*

*Biochemistry*

As shown in the tabulated results in Table (1), NDEA administration elicits significant elevation (P<0.05) in serum ALP, AST and ALT enzymes activity relative to the negative control group. Also, the findings in Table (2) indicated that there is significant upregulation (P<0.05) in serum levels of urea and creatinine in NDEA- administered group in respect with the negative control group. The enhancement of liver enzymes activity (ALP, AST and ALT) in serum of rats subjected to NDEA administration indicates the incidence of hepatocellular damage (Bansal et al., 2005). The amount of these cellular enzymes in the blood could be attributed to the leakage of these enzymes from the cytoplasm into the blood circulation after alteration in plasma membrane integrity and /or permeability. Liver enzymes like AST and ALT, have been reported to be increased in HCC condition (Saharawi et al., 2009). Another key hepatic marker enzyme is ALP whose enhanced activity in serum evidences the pathological changes in bile flow. Reporter assays indicated that the dividing cells shed more of ALP as they are localized in the bile canalicular plasma membrane (Frederiks et al., 1990). Therefore, the overproduction of this enzyme in tumor cells may potentiate the permeability of the cellular membrane leading to such drastic increase of this enzyme in serum (Revathi et al., 2013). NDEA metabolized by cytochrome p450 generates a highly reactive free radical, and initiates lipid peroxidation of the cell membrane of the endoplasmic reticulum and causes a chain reaction. These reactive oxygen species can cause oxidative damage in DNA, proteins and lipids (Archer, 1989; Vitaglione et al., 2004). NDEA administration to rats lead to a marked elevation in the levels of serum BUN and creatinine which is indicative of kidney damage, as previously reported (Khan et al., 2001; Atakisi et al., 2013). 

**Figure 1:**
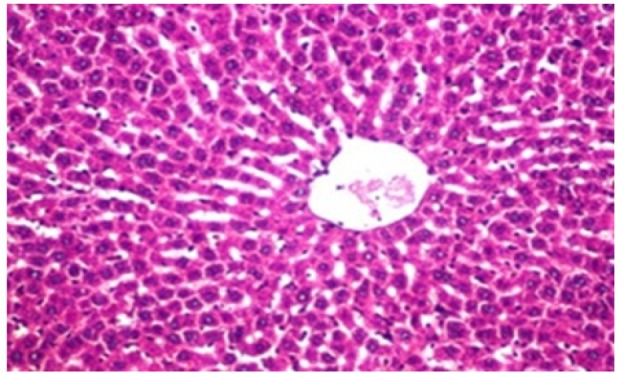
Photomicrograph of Liver Tissue Section of Rat in the Negative Control Group Showing Normal Histological Structure of the Central Vein and Surrounding Hepatocytes in the Parenchyma. (H&E x40)

**Figure 2:**
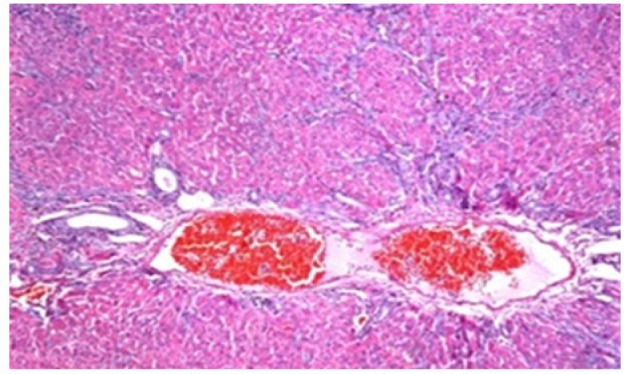
Photomicrograph of Liver Tissue Section of Rat in NDEA-Administered Group (HCC Group) Showing Sever Congestion in the Portal Vein with Dysplasia and Anaplasia of the Hepatocytes in Hepatic Parenchyma. (H&E x16)

**Table 1:** Influence of Treatment with Compounds **5, 4** and **3** on Serum ALP, AST and ALT Activity in NDEA - Challenged Rats

Groups	ALP U/L	AST U/L	ALT U/L
Negative control	100.52 ± 1.43	39.50 ±1.13	25.57 ± 0.84
HCC	143.12± 1.56 ^a^*	90.76 ±1.06 ^a^*	45.050 ± 0.93 ^a^*
HCC + DOXO	142.62 ±1.60	86.71 ± 4.59	41.72 ±1.64
HCC + comp **5**	110.27 ± 1.09 ^b^*	43.21 ± 0.46 ^b^*	28.31 ±.92 b*
HCC + comp **4**	114.07 ± 1.22 ^b^*	43.95 ±.97 ^b^*	25.67 ±1.06 ^b^*
HCC + comp **3**	120.37 ± 0.80 ^b^*	44.75. ± 0.80 ^b^*	27.82± 0.44 ^b^*

*Significant difference at P<0.05; ^a^, Significant difference versus the negative control group, ^b^, Significant difference versus the HCC group

**Table 2:** Influence of Treatment with Compounds **5, 4** and **3** on Serum Urea and Creatinine Levels in NDEA-Challenged Rats

Groups	Urea g/dl	Creatinine g/dl
Negative control	37.63 ±1.57	0.912 ± 0.05
HCC	64.47 ± 3.12^ a^*	1.60 ± 0.07 ^a^*
HCC + DOXO	59.50 ± 3.62	1.58 ±0 .032
HCC + comp **5**	42.60 ± 2.39 ^b^*	0.97 ± 0.05 ^b^*
HCC + comp **4**	45.51 ± 3.97 ^b^*	1.02 ±0.03 ^b^*
HCC + comp **3**	43.66 ±1.61 ^b^*	0.98 ± 0.03 ^b^*

* Significant difference at P<0.05; ^a^, Significant difference versus the negative control group, ^b^, Significant difference versus the HCC group

**Table 3:** Influence of Treatment with Compounds **5, 4 **and **3** on Serum AFP, ENG, LCN-2 and HSP-70 Levels in NDEA-Challenged Rats

Groups	AFP ng/ml	ENG ng/ml	LCN-2 ng/ L	HSP-70 ng/ L
Negative control	33.07± 2.18	4.01±.06391	231.00 ±9.53	32.65 ± .34
HCC	41.53± 0.57 ^a^*	13.06 ± 0.31 ^a^*	374.25 ±3.66 ^a^*	66.26 ± .79 ^a^*
HCC + DOXO	37.90 ± 0 .49 ^b^*	8.63 ± 0.31 ^b^*	262.37 ±5.51 ^b^*	37.05 ±1.13 ^b^*
HCC + comp **5**	36.37± 0 .97 ^b^*	5.10 ± 0.20 ^b^*	235.50 ±4.31 ^b^*	35.50 ±1.79 ^b^*
HCC + comp **4**	38.87± 0 .23 ^b^*	7.00 ± 0.42 ^b^*	257.20 ±14.23 ^b^*	39.95 ±1.23 ^b^*
HCC + comp **3**	37.83± 0.54 ^b^*	7.05 ± 0.14 ^b^*	267.40 ±16.33 ^b^*	37.45 ± 1.10 ^b^*

* Significant difference at P<0.05,^a^, Significant difference versus the negative control group; ^b^, Significant difference versus the HCC group

**Figure 3:**
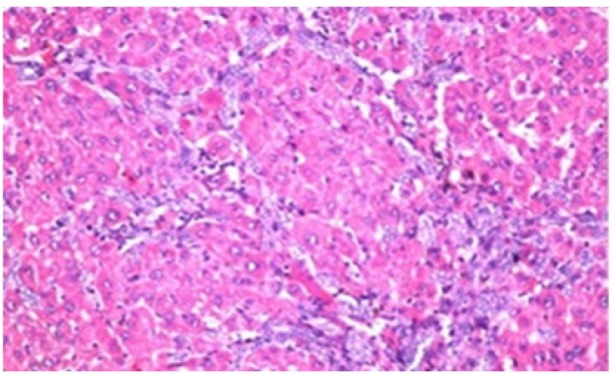
Photomicrograph of Liver Tissue Section of Rat in NDEA-Administered Group (HCC Group) Showing the Magnification of (Figure 2) to Identify the Dysplasia of the Hepatocytes and Irregular Arrangement. (H&E x40)

**Figure 4:**
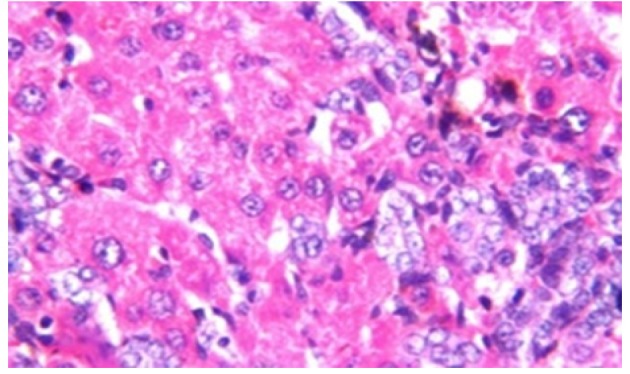
Photomicrograph of Liver Tissue Section of Rat in NDEA-Administered Group (HCC Group) Showing the Magnification of (Figure 3) to Identify the Anaplastic Hepatocytes with Prominent Nucleolus and Pleomorphism. (H&E x80)

**Figure 5:**
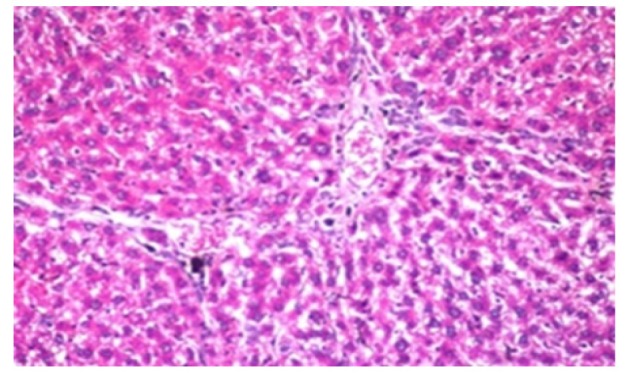
Photomicrograph of Liver Tissue Section of Rat Administered NDEA and Treated with DOXO Showing Fine Fibrosis with Brown Pigment in between the Hepatocytes. (H&E x40)

**Figure 6:**
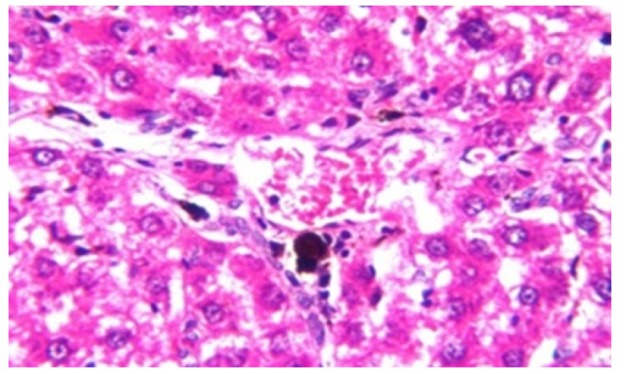
Photomicrograph of Liver Tissue Section of Rat Administered NDEA and Treated with DOXO Showing the Magnification of (Figure 5) to Identify the Brown Pigment between the Hepatocytes. (H&E x80)

**Figure 7:**
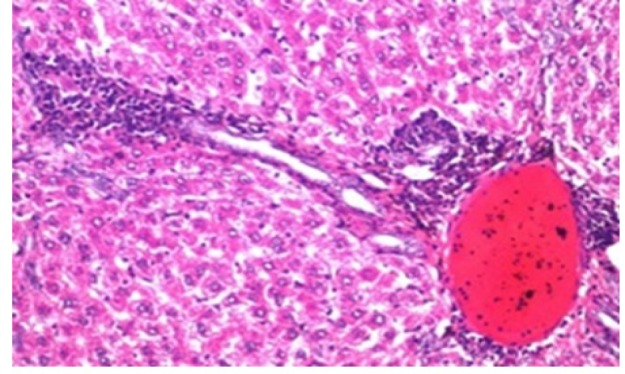
Photomicrograph of Liver Tissue Section of Rat Administered NDEA and Treated with Compound **5** Showing Massive Number of Inflammatory Cells Infiltration in the Portal Area with Congestion in Portal Vein. (H&E x40)

**Figure 8:**
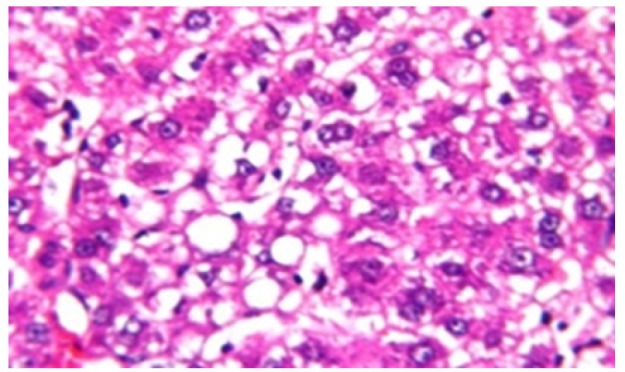
Photomicrograph of Liver Tissue Section of Rat Administered NDEA and Treated with Compound **5** Showing Focal Area of Fatty Change in the Hepatic Cells. (H&E x80)

**Figure 9:**
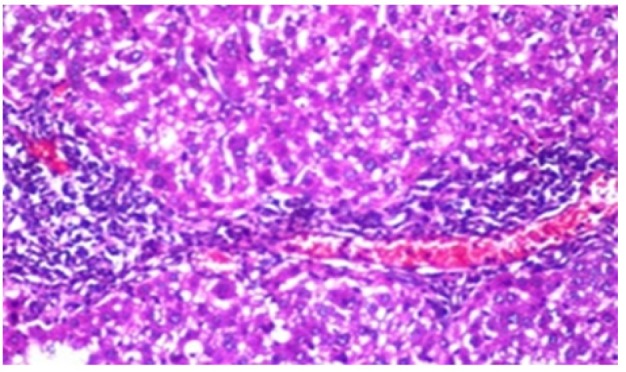
Photomicrograph of Liver Tissue Section of Rat Administered NDEA and Treated Compound **4** Showing Massive Number of Inflammatory Cells Aggregation in the Portal Area. (H&E x40)

**Figure 10:**
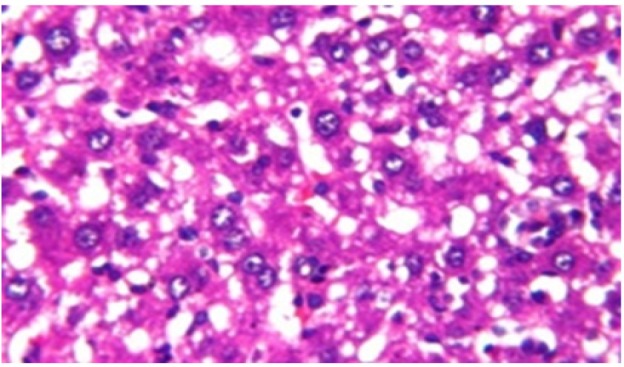
Photomicrograph of Liver Tissue Section of Rat Administered NDEA and Treated with Compound **4 **Showing Fatty Change in Some of the Hepatocytes in the Parenchyma. (H&E x80)

**Figure 11:**
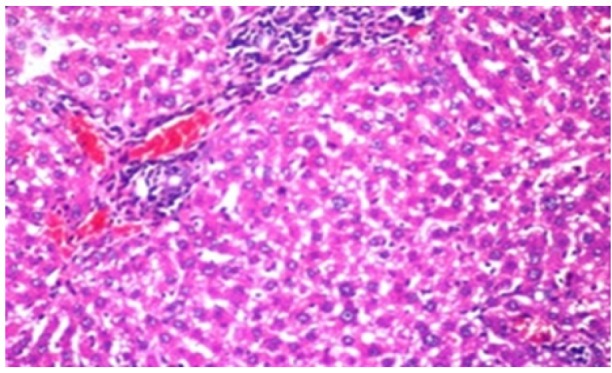
Photomicrograph of Liver Tissue Section of Rat Administered NDEA and Treated with Compound **3 **Showing Inflammatory Cells Infiltration in the Portal Area. (H&E x40)

**Figure 12:**
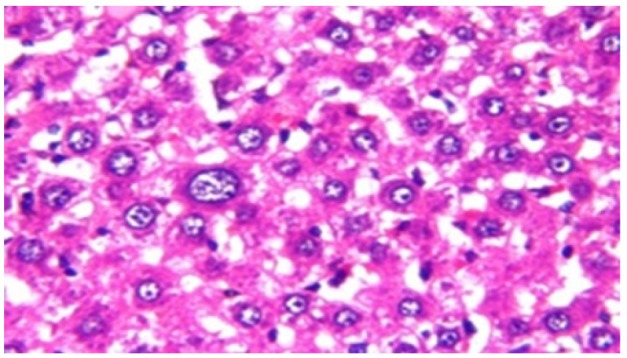
Photomicrograph of Liver Tissue Section of Rat Administered NDEA and Treated with Compound **3 **Showing Fatty Change in Hepatocytes with Karyomegaly in the Others as well as Kupffer Cells Proliferation in between. (H&E x80)

**Figure 13:**
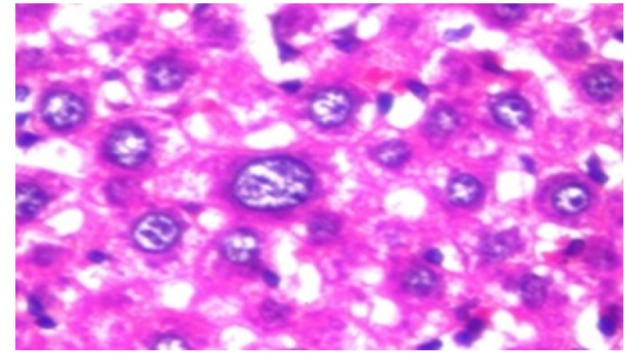
Photomicrograph of Liver Tissue Section of Rat Administered NDEA and Treated with Compound **3** Showing the Magnification of (Figure 12) to Identify the Fatty Changes and Karyomegaly in the Hepatocytes.(H&E x160)

Treatment with DOXO produces insignificant change (P˃0.05) in serum ALP, AST and ALT enzymes activity when compared with HCC group Table (1). Likewise, DOXO causes insignificant change (P˃0.05) in serum urea and creatinine levels versus HCC group Table (2). Previous studies revealed that DOXO- treated animals experience significant increase in the activity of ALT and AST enzymes in serum when compared with the control counterparts (Adejuwon et al., 2008; Kusuzaki et al., 2000). These research groups explained the higher activity of AST and ALT as drastic conditions caused by the toxic impact of DOXO accumulation in the liver which in turn might provoke hepatocellular inflammation and damage with consequent increase in the permeability of hepatic cells membrane. The results of Ezzat et al., (2005) showed that serum ALP activity is significantly increased upon treatment of mice with DOXO versus the controls. DOXO is also known as nephrotoxic candidate that produces chronic progressive glomerular disease manifested by increased plasma creatinine and urea levels in association with extensive glomerular lesions, tubular dilatation, vacuolization of renal glomeruli, protein deposits in tubular lumen and stromal fibrosis (Injac et al., 2008; Ahmed et al., 2013). These findings explained the present results of the insignificant change in serum urea and creatinine levels in HCC group treated with DOXO in comparison with HCC group. 

The treatment with compounds **5, 4 **or **3** evokes significant reduction (P<0.05) in serum ALP, AST and ALT enzymes activity versus HCC group Table (1). Similarly, the treatment with compounds **5, 4** or **3 **induces significant regression (P<0.05) in serum urea and creatinine levels relative to HCC group Table (2). The significant decline in serum ALP, AST and ALT enzymes activity as well as urea and creatinine serum levels in HCC bearing rats treated with compound **5** (pyrazole derivative) comes in line with the results obtained by Shabbir et al., (2009) who recorded insignificant change in serum AST, ALT, urea and creatinine values in pyrazole compound- treated rats in comparison with the controls. Also, no significant change in ALP activity in serum has been registered upon treatment of rats with pyrazole compound relative to the controls (Eidi et al., 2006; Kesari et al., 2007). These data emphasized the absence of hepatotoxicity or nephrotoxicity regarding pyrazole derivative. The significant reduction in serum ALP, AST and ALT enzymes activity in association with the significant depletion in serum urea and creatinine levels in HCC bearing rats treated with compound **4** (thiazine derivative) go hand in hand with the study of Shabbir et al., (2009) which showed a decreased serum activity of liver enzymes in animals treated with thiazine derivatives. Also, this research group commented that the thiazine derivatives do not produce any signs of hepatotoxicity or nephrotoxicity. The significant inhibition in serum ALP, AST and ALT enzymes activity in combination with significant suppression in serum urea and creatinine levels in HCC bearing rats treated with compound **3** (pyrimidine derivative) are in harmony with the previous findings of Vyshtakalyuk et al., (2016) who recorded a statistically insignificant decrease in ALT, AST and ALP enzymes activity in the pyrimidine derivatives-treated rats in comparison with the control counterparts. Lewis (1912) has reported about the behavior of the hydantoin nucleus, a structure similar to the pyrimidine. This investigator reported that hydantoin has no toxic effect on kidney functions. These results emphasize that there are no toxic effects on liver or kidney with regard to pyrimidine derivatives. 

The data in Table (3) revealed that NDEA administration leads to significant enhancement (P<0.05) in serum AFP, ENG, LCN-2 and HSP-70 levels in comparison with the negative control group. AFP is the most common biochemical marker for the diagnosis of HCC and the persistent amplification of AFP is considered as a risk factor for developing HCC (Mohamed et al., 2015). The significant upregulation of serum AFP in HCC group in the present study is comparable to that of Ahmed et al., (2015). It has been found that AFP gene expression is significantly upregulated in NDEA administered rats and it was considered as a strong indicator for the onset of HCC. The probable mechanism for the reinitiation of AFP synthesis by neoplastic hepatocytes involves either enhanced *AFP* gene transcription or post-translational modification inducing AFP production (Yang and Poon, 2008). 

Endoglin (CD105) is an endothelial cell biomarker that reflects the endothelial cells proliferation status as an angiogenic molecule (Ho et al., 2005). The significant amplification of serum ENG level in HCC group in the current research comes in line with that of Song et al. (2013). It has been demonstrated that ENG is positively stained by CD105 in a subset of microvessels of HCC stain providing significant prognostic information about HCC (Nassiri et al., 2011). Moreover, a significant positive correlation between serum ENG level and ALP, AST and ALT, total bilirubin as well as AFP levels has been demonstrated in HCC patients by Mohamed et al., (2015).

Lipocalin-2 (LCN-2) expression has been observed in most tissues, and its synthesis is induced in epithelial cells during inflammation. Increasing evidence suggests that inflammation is closely related to tumorigenesis (Wang et al., 2013). Also, LCN-2 has been found to be involved in different vital processes of the cells like the innate immune response, differentiation, carcinogenesis and cell survival (Gehrmann et al., 2014). Thus, the significant elevation in serum LCN-2 level in HCC group in the present setting confirmed the onset of HCC by NDEA administration and the implication of LCN-2 in the carcinogenic process. Many tumor entities including HCC have been found to overexpress the common-stress-inducible member of HSPs family (HSP70), that is existed on the surface of the tumor cell and secreted into the extracellular microenvironment (Bayer et al., 2014). Our previous study Ahmed et al., (2015) proved significant upregulation in HSP70 gene expression level in the liver tissue of NEDA administered rats. This finding supports the hypothesis that the great soluble HSP70 serum level stems from viable tumor cells that actively release HSP70 in lipid vesicles and not by inflamed liver necrotic tissue (Zhang et al., 2006). The current results also are in great agreement with this hypothesis.

DOXO treatment in HCC group produces significant suppression (P<0.05) in serum AFP, ENG, LCN-2 and HSP-70 levels relative to HCC group Table 3. Regarding the blunted serum level of AFP in DOXO –treated rats in the present study, the finding of Morsi et al., (2006) demonstrated that HCC patients treated with Adriamycin (DOXO) display a significant reduction in AFP serum level. Such depletion in AFP by Adriamycin could be attributed to the ability of this drug to reduce tumor mass resulting in the decreased AFP synthesis by the cancer cells. This explanation is greatly supported by Kusuzaki et al., (2016) who emphasized the potentiality of anti-tumor activity of DOXO.

The significant suppression of ENG serum level upon treatment of HCC bearing rats with DOXO in the present study could be explained by the study of Zanini et al., (2007) which proved that DOXO with the standard dose inhibits tumor growth via its antiangiogenic effect in vivo. Moreover, DOXO has been shown to cause DNA damage and eventually induces programmed cell death in many tumor cells. Furthermore, DOXO has been found to increase apoptosis and decrease proliferation of the endothelial cell population which in turn lead to stopping of angiogenesis and tumor cell proliferation (Gupta et al., 2013). 

The significant blunting of LCN-2 serum level in HCC bearing rats treated with DOXO in the current investigation could be ascribed to the antitumor efficacy of DOXO which results in decreasing tumorigenesis related proteins (Pietras et al., 2005). DOXO could activate apoptosis by inducing proteolytic processing of Bcl-2 family and simultaneously decrease oxidative stress by influencing ROS damage in MCF-7 and MDA-MB-231 cells (Ferreto and Calaf, 2016).

The recorded significant depletion of serum HSP-70 level in HCC bearing rats treated with DOXO in the current setting could be explained by the anticancer potential of DOXO which is responsible for the limitation of tumor growth and alleviation of tumor stress on liver tissue. It has been reported that the treatment with DOXO significantly downregulate *HSPgp96* gene expression level in liver tissue of HCC bearing rats (Williams et al., 2001). 

Treatment of HCC group with compounds **5, 4** or **3** yields significant depletion in serum AFP, ENG, LCN-2 and HSP-70 levels when compared with HCC group. Several types of steroids have been modified to be cytotoxic and cytostatic (antiproliferative) anticancer candidates (Tzanetou et al., 2012). Also, many steroids were among the first molecules identified as antiangiogenic agents (Labbozzetta et al., 2009). The potency of such molecules was manifested mainly by their capability to prevent the growth of new blood vessels for angiogenesis (Badshah and Naeem, 2016). The study on the interactions of steroid with vascular endothelial cells indicated that it binds with cell membranes, decreases the membrane Na_/H_ exchanger and acts as a calmodulin chaperone. These initial actions seem to suppress many critical stages in angiogenesis, like blockade of mitogen- induced actin polymerization, cell–cell adhesion and cell migration, resulting in inhibition of endothelial cell proliferation (Christodoulou et al., 2010). Moreover, Anzaldi et al., (2009) proved that the antiproliferative and proapoptotic properties of short heterocyclic derivatives are deeply associated with their heterocyclic moiety (pyrazole, isoxazole and pyrimidine). 

The antitumor activity of compound **5** might be ascribed to the presence of pyrazole ring beside the steroid moiety as it was reported that pyrazole derivatives, strongly suppress the growth of human colon cancer cells and promote programmed cell death by enhancing the proportion of sub-G1 presenting apoptotic cells, reducing cell cycle arrest at the G2/M phase and inhibiting the proliferation of the cells (Badshah and Naeem, 2016). Moreover, pyrazoles that display anticancer potential have been studied by Christodoulou et al., (2010). These investigators mentioned that different N-substituted pyrazoles are implemented as antileukemic, antitumor, antiproliferative, anti-angiogenic, DNA interacting, proapoptotic and antitubulin agents. Besides, these candidates are able to exhibit marked anti-cancer effects via the suppression of many enzymes, proteins and receptors which have a key role in cell division. In addition, some pyrazole compounds have been characterized by their powerful antiproliferative activity in vitro and antitumor activity in vivo (Ribatti, 2014). Aside from these actions, the antiangiogenic agents are distributed by their actions into two main classes (a) indirect one that leads to blocking the activity of angiogenic molecules, or the expression of their receptors on the endothelial cells, and (b) the direct action that results in affection on endothelial cell functions or survival directly (Tzanetou et al., 2014). Thus, the development of specific antiangiogenic drugs represents an attractive therapeutic strategy for the treatment of cancer. Pyrazoles has been found to downregulate the expression of hypoxia-inducible factor 1-alpha (HIF-1α) and vascular endothelial growth factor (VEGF) as well as human umbilical vein endothelial cells (HUVEC) tube formation and migration, thereby they could inhibit the angiogenesis process (Ferreira et al., 2013).

Concerning the antitumor potential of compound **4**, in addition to the steroid nucleus, Ferreira and his colleagues reported that thiazine derivatives display anticancer activity against leukemia cells (Ferreira et al., 2013). Earlier study proved that thiazine derivatives inhibit different types of blood cancer cells due to antitumor, antiproliferative and antiinflammatory properties (Andres and Arthur, 2004). The DNA fragmentation (tumor cell death) has been found to be produced by several aromatic groups attached to the thiazine derivatives that probably intercalate with the targeted DNA leading to DNA fragmentation and ultimately cell death. Besides, thaizine derivatives have been used to stop lung, colon and other types of progressing cancers without toxic effect on normal body cells (Juszczak et al., 2016). It was also emphasized that the activation of caspase cascade occurs, along with the disparity in intracellular calcium ions and aberration in the main cell functional organelles upon treatment of cancer cells with thiazine. Thiazine derivatives could regulate DNA synthesis and the different cell growth stages by inhibiting p38 kinase and cyclin D1 enzymes activity (Jele´n et al., 2015).

With respect to the impact of compound **3** on the circulating markers that are related to the onset of HCC in the present investigation, Apart from steroid molecule, large number of structurally pyrimidines have been reported to exhibit substantial anticancer activity in vitro and in vivo (Shi et al., 2007). Some pyrimidine derivatives carrying the biologically active thione moiety at 2-position displayed promising anti-breast cancer activity higher than that of DOXO. Other, pyrimidine derivatives could impair glioma tumor adhesion, migration, and invasion (Kurio et al., 2011) and suppress oral squamous cell carcinoma growth and angiogenesis in a xenograft mouse model (Kurio et al., 2012). The antiproliferative and apoptotic activities of pyrimidine derivatives *in vitro* in human A2780 - A549 and murine P388 tumor cells have been proved (Golubovskaya et al., 2008). Moreover, pyrimidine derivatives are capable of accomplishing the detachment and apoptosis of breast cancer (Kurio et al., 2012). 

*Histopathology*

Histological investigation of liver tissue section of rat in the negative control group showed normal structure of central vein and the surrounding hepatocytes in the parenchyma (Figure 1). Concerning rats administered NDEA, the microscopic examination of liver tissue sections showed sever congestion in the portal vein associated with dysplasia with irregular histological arrangement and anaplasia of the hepatocytes all over the hepatic parenchyma (Figures 2 and 3). The anaplastic hepatocytes showed prominent nucleoli and pleomorphism (Fig. 4). These findings converge with those observed in the studies of Seufi et al., (2009) and Darwish and El-Boghdady., (2011).Microscopic investigation for liver tissue sections of rat administered NDEA and treated with DOXO showed fine fibrosis with brown pigment in between the hepatocytes (Figures 5 and 6). These observations fit with those obtained by El-Sayyad et al., (2009). Microscopic investigation of liver tissue sections of rat administered NDEA and treated with compound **5** showed massive number of inflammatory cells infiltration in the portal area (Figure 7), associated with focal area of fatty change in the hepatocytes all over the parenchyma (Figure 8). These finding are comparable to those of Majhool et al., (2016). Microscopic investigation for liver tissue sections of rat administered NDEA and treated with compound **4** showed inflammatory cells aggregation in the portal area (Figure 9), paralleled by fatty changes in the hepatocytes in the parenchyma (Figure 10). These observations are in compliance with those demonstrated by Khalilullah et al., (2011). Microscopic investigation of liver tissue sections of rat administered NDEA and treated with compound** 3** showed inflammatory cells infiltration in the portal area (Figure 11) while the hepatic parenchyma showed fatty changes in some of the hepatocytes and karyomegalic nuclei in the others as well as diffuse Kupffer cells proliferation in between (Figures 12 and 13). These results corroborate the findings of Aziz et al., (2016).

In conclusion, this study introduced a facile synthesis of new hybrid steroid derivatives in moderate to high yield, via MCRs and emphasized the importance of incorporating heterocyclic moieties to the steroid nucleus in order to produce effective anticancer agents. Compounds **3, 4 **and **5** containing dithioxopyrimidine, thioxothiazine and pyrazole moieties, respectively, incorporated to pyridosteroid nucleus displayed promising antitumor activity, antiangiogenic potential and anti-inflammatory effect in HCC rat model. Interestingly, compounds **3, 4** and **5** exhibited superior anticancer properties when compared with the reference drug (DOXO). 

## References

[B1] Adejuwon AA, Adokiye SB (2008). Protective effect of the aqueous leaf and seed extract of Phyllanthus amarus on gentamicin and acetaminophen induced nephrotoxic rats. J Ethnopharmacol.

[B2] Ahmed F, Uroo JA, Karim AA (2013). Protective effects of Ficus racemosa stem bark against doxorubucin-induced renal and testicular toxicity. Pharmacogen Mag.

[B3] Andres AC, Arthur IC (2004). Oxidative stress toxicology, and pharmacology of CYP2E1. Annu Rev Pharmacol.

[B4] Anzaldi M, Macciò C, Mazzei M (2009). Antiproliferative and proapoptotic activities of a new class of pyrazole derivatives in HL-60 cells. Chem Biodivers.

[B5] Archer MC (1989). Mechanisms of action of N-nitroso compounds. Cancer Surv.

[B6] Armitage P, Berry G (1987). Comparison of several groups: Statistical method in medical research.

[B7] Atakisi O, Atakisi E, Ozcan A (2013). Protective effect of omega-3 fatty acids on diethyl nitrosamine toxicity in rats. Eur Rev Med Pharmacol Sci.

[B8] Aziz MA, Rabah AT, Serya DS (2016). Discovery of potent VEGFR-2 inhibitors based on furopyrimidine and thienopyrimidne scaffolds as cancer targeting agents. Sci Rep.

[B9] Badshah SL, Naeem A (2016). Bioactive thiazine and benzothiazine derivatives: green synthesis methods and their medicinal importance. Molecules.

[B10] Bansal AK, Bansal M, Soni G (2005). Protective role of Vitamin E pre-treatment on N-nitrosodiethylamine induced oxidative stress in rat liver. Chem Biol Interact.

[B11] Barnes JM, Paget GE (1965). Mechanisms of toxic action. Prog Med Chem.

[B12] Bayer C, Liebhardt ME, Schmid TE (2014). Validation of heat shock protein 70 as a tumor-specific biomarker for monitoring the outcome of radiation therapy in tumor mouse models. Int J Radiat Oncol Biol Phys.

[B13] Brown MS, Goldstei JL (1985). A receptor mediated pathway for cholesterol homeostatis. Nobel Lecture. Stockholm.

[B15] Christodoulou MS, Liekens S, Kasiotis KM (2010). Novel pyrazole derivatives: synthesis and evaluation of anti-angiogenic activity. Bioorg Med Chem.

[B16] Chubb FL, Edward F (1981). The reaction between carbon disulfide and some α-methylaminonitriles The supposed isomerism of 4,4-dialkyl-5-imino-3-methylthiazolidine-2-thiones. Can J Chem.

[B17] Darwish HA, El-Boghdady NA (2011). Possible involvement of oxidative stress in diethylnitrosamine induced hepatocarcinogenesis: chemopreventive effect of curcumin. J Food Biochem.

[B18] Eidi A, Eidi M, Esmaeili E (2006). Antidiabetic effect of garlic (Allium sativum L) in normal and streptozotocin-induced diabetic rats. Phytomedicine.

[B19] El-Far M, Elmegeed GA, Eskander EF (2009). Novel modified steroid derivatives of androstanolone as chemotherapeutic anti-cancer agents. Eur J Med Chem.

[B20] El-Sayyad H, Ismail MF, Shalaby FM (2009). Histopathological effects of cisplatin, doxorubicin and 5-fluorouracil (5-FU) on the liver of male albino rats. Int J Biol Sci.

[B21] Ezzat S, Abdel-Hamid M, Eissa SA (2005). Associations of pesticides, HCV, HBV, and hepatocellular carcinoma in Egypt. Int J Hyg Environ Health.

[B22] Ferreira M, Assunção LS, Filippin-Monteiro FB (2013). Synthesis of 1, 3-thiazine-2,4-diones with potential anticancer activity. Eur J Med Chem.

[B23] Ferreto NP, Calaf GM (2016). Influence of doxorubicin on apoptosis and oxidative stress in breast cancer cell lines. Int J Clin Oncol.

[B24] Frederiks WM, Van Noorden CJ, Aronson DC (1990). Quantitative changes in acid phosphatase, alkaline phosphatase and 5’-nucleotidase activity in rat liver after experimentally induced cholestasis. Liver.

[B25] Fuente A, Reyes M, Alvarez YM (2005). 1H and 13C NMR spectral assignment of androstane derivatives. Magn Reson Chem.

[B26] Gehrmann M, Cervello M, Montalto G (2014). Heat shock protein 70 serum levels differ significantly in patients with chronic hepatitis, liver cirrhosis, and hepatocellular carcinoma. Front Immunol.

[B27] Golubovskaya VM, Virnig C, Cance WG (2008). TAE226-induced apoptosis in breast cancer cells with overexpressed Src or EGFR. Mol Carcinogen.

[B28] Gupta AB, Kumar S, Arvind S (2013). Current status on development of steroids as anticancer agents. J Steroid Biochem Mol Biol.

[B29] Gupta R, Pathak D, Jindal DP (1996). Synthesis and biological activity of azasteroidal [3,2-c]- and [17,16-c] pyrazoles. Eur J Med Chem.

[B30] He Q, Jiang D (1999). A novel aminosteroid is active for proliferation inhibition and differentiation induction of human acute myeloid leukaemia HL-60 cells. Leuk Res.

[B31] Ho JW, Poon RT, Sun CK (2005). Clinicopathological and prognostic implications of endoglin (CD105) expression in hepatocellular carcinoma and its adjacent non-tumorous liver. World J Gastroenterol.

[B32] Hosking D, Chilvers CE, Christiansen C (1998). Prevention of bone loss with alendronate in postmenopausal women under 60 years of age Early postmenopausal intervention cohort study group. J N Engl J Med.

[B33] Hosny G, Farahat N, Tayel H (2008). Ser-249 TP53 and CTNNB1 mutations in circulating free DNA of Egyptian patients with hepatocellular carcinoma versus chronic liver diseases. Cancer Lett.

[B34] Injac R, Strukelj B (2008). Recent advances in protection against doxorubicin-induced toxicity. Technol Cancer Res Treat.

[B35] Jele´n M, Pluta K, Zimecki M (2015). 6-substituted 9-fluoroquino (3,2-b)benzo(1,4)thiazines display strong antiproliferative and antitumor properties. Eur J Med Chem.

[B36] Jemal A, Bray F, Center MM (2011). Global cancer statistics. CA Cancer J Clin.

[B37] Juszczak M, Walczak K, Matysiak J (2016). New derivative of 2-(2, 4-dihydroxyphenyl) thieno-1,3-thiazin-4-one (BChTT) elicits antiproliferative effect via p38-mediated cell cycle arrest in cancer cells. Bioorg Med Chem.

[B38] Kesari AN, Kesari S, Singh SK (2007). Studies on the glycemic and lipidemic effect of Murraya koenigii in experimental animals. J Ethnopharmacol.

[B39] Kew MC (2014). Hepatocellular carcinoma: epidemiology and risk factors. J Hepatocell Carcinoma.

[B40] Khalilullah H, Khan Sh, Ahsan MJ (2011). Synthesis and antihepatotoxic activity of 5-(2,3-dihydro-1,4-benzodioxane-6-yl)-3-substituted-phenyl-4,5-dihydro-1H-pyrazole derivatives. Bioorg Med Chem Lett.

[B41] Khan N, Sharma S, Alam A (2001). Tephrosia pursuer ameliorates N-diethyl nitrosamine and potassium bromated-mediated renal oxidative stress and toxicity in Wistar rats. Pharmacol Toxicol.

[B42] Kirk GD, Lesi OA, Mendy M (2004). The Gambia liver cancer study: infection with hepatitis B and C and the risk of hepatocellular carcinoma in West Africa. Hepatology.

[B43] Kurio N, Shimo T, Fukazawa T (2011). Anti-tumor effect in human breast cancer by TAE226, a dual inhibitor for FAK and IGF-IR in vitro and in vivo. Exp Cell Res.

[B44] Kurio N, Shimo T, Fukazawa T (2012). Anti-tumor effect of a novel FAK inhibitor TAE226 against human oral squamous cell carcinoma. Oral Oncol.

[B45] Kusuzaki K, Shinjo H, Murata H (2000). Relationship between doxorubicin binding ability and tumor volume decrease after chemotherapy in adult malignant soft tissue tumors in the extremities. Anticancer Res.

[B46] Labbozzetta M, Baruchello R, Marchetti P (2009). Lack of nucleophilic addition in the isoxazole and pyrazole diketone modified analogs of curcumin, implications for their antitumor and chemosensitizing activities. Chem-Biol Interact.

[B47] Latham KA, Zamora A, Drought H (2003). Estradiol treatment redirects the isotype of the autoantibody response and prevents the development of autoimmune arthritis. J Immunol.

[B48] Lewis HB (1912). The behavior of some hydantoin derivatives in metabolism Hydantoin and Ethyl Hydantoate. J Biol Chem.

[B49] Li X, Singh SM, Cote J (1995). Synthesis and in vitro evaluation of 4-substituted N-(1, 1- dimethylethyl)-3-oxo-4-androstene-17b- carboxamides as 5a-reductase inhibitors and antiandrogens. J Med Chem.

[B50] Majhool AB, Zenad Kh H (2016). Histopathological effects of different doses of anabolic androgenic steroid (Sustanon) on liver and testis of male rats. JABR.

[B51] Mohamed NR, Elmegeed GA, Abdelhalim MM (2010). Facile synthesis and in vitro cytotoxic evaluation of novel thiadiazole, pyrazole, and dithiole-androstane derivatives. J Phosphours Sulfur Silicone.

[B52] Mohamed NR, Elmegeed GA, Abd-ElMalek HA (2005). Synthesis of biologically active steroid derivatives by the utility of Lawesson’s reagent. Steroids.

[B53] Mohamed RA, Maghraby HM, Abd El Salam EM (2015). Evaluation of serum endoglin as noninvasive marker in hepatocellular carcinoma. EJIM.

[B54] Morsi MI, Hussein AE, Mostafa M (2006). Evaluation of tumor necrosis factor-α(TNF-α), soluble P-selectin (sP-Selectin), gamma-glutamyl transferase (GGT), glutathione Stransferase Pi (GST-Pi) and alpha-fetoprotein (AFP) in patients with hepatocellular carcinoma before and during chemotherapy. Br J Biomed Sci.

[B55] Nassiri F, Cusimano MD, Scheithauer BW (2011). Endoglin (CD105): a review of its role in angiogenesis and tumor diagnosis, progression and therapy. Anticancer Res.

[B56] Pietras RJ, Weinberg OK (2005). Antiangiogenic steroids in human cancer therapy. Evid-Based Compl Al Med.

[B57] Rahman AM, Yusuf SW, Ewer MS (2007). Anthracycline-induced cardiotoxicity and the cardiac-sparing effect of liposomal formulation. Int J Nanomedicine.

[B58] Revathi R, Manju V (2013). The effects of umbelliferone on lipid peroxidation and antioxidant status in diethylnitrosamine induced hepatocellular carcinoma. J Acute Med.

[B59] Ribatti D (2014). The discovery of angiogenic growth factors: the contribution of Italian scientists. Vasc Cell.

[B60] Seufi A, Safinz S, Ibrahim S (2009). Preventive effect of the flavonoid, quercetin, on hepatic cancer in rats via oxidant/antioxidant activity: molecular and histological evidences. J Exp Clin Canc Res.

[B61] Shaarawy SM, Tohamy AA, Elgendy SM (2009). Protective effects of garlic and silymarin on NDEA-induced rats hepatotoxicity. Int J Biol Sci.

[B62] Shabbir A, Shahzad M, Ali A (2016). Discovery of new benzothiazine derivative as modulator of pro- and anti-inflammatory cytokines in rheumatoid arthritis. Inflammation.

[B63] Shi Q, Hjelmeland AB, Keir ST (2007). A novel low-molecular weight inhibitor of focal adhesion kinase, TAE226, inhibits glioma growth. Mol Carcinog.

[B64] Smolyar NN, Yutilov YM (2009). Reduction of 2-amino-3- and -5-nitropyridine derivatives with hydrazine hydrate. Russ J Org Chem.

[B65] Song Y, Jin S, Cui LH (2013). Immunomodulatory effect of Stichopus japonicus acid mucopolysaccharide on experimental hepatocellular carcinoma in rats. Molecules.

[B66] Sreepriya M, Bali G (2005). Chemopreventive effects of embelin and curcumin against N- nitrosodiethylamine/ phenobarbital-induced hepatocarcinogenesis in Wistar rats. Fitoterapia.

[B67] Tantawy MA, Mohamed SN, Elmegeed GA (2017). Auspicious role of the steroidal heterocyclic derivatives as a platform for anti-cancer drugs. Bioorg Chem.

[B69] Tzanetou E, Liekens S, Kasiotis KM (2014). Antiproliferative novel isoxazoles: modeling, virtual screening, synthesis, and bioactivity evaluation. Eur J Med Chem.

[B70] Tzanetou E, Liekens S, Kasiotis KM (2012). Novel pyrazole and indazole derivatives: synthesis and evaluation of their anti-proliferative and anti-angiogenic activities. Arch Pharm (Weinheim).

[B71] Varmus H (2006). The new era in cancer research. Science.

[B72] Visvanathan K, Davidson NE 2003) Aromatase inhibitors as adjuvant therapy in breast cancer. Oncology (Williston Park).

[B73] Vitaglione P, Morisco F, Caporaso N (2004). Dietary antioxidant compounds and liver health. Crit Rev Food Sic Nutr.

[B74] Vyshtakalyuk AB, Nazarov NG, Zobov VV (2016). Evaluation of the hepatoprotective effect of L-ascorbate 1-(2 hydroxyethyl)-4,6-dimethyl-1,2-dihydropyrimidine- 2-one upon exposure to carbon tetrachloride. B Exp Biol Med.

[B75] Wang YP, Yu GR, Lee MJ (2013). Lipocalin-2 negatively modulates the epithelial-to- mesenchymal transition in hepatocellular carcinoma through the epidermal growth factor (TGF beta1)/Lcn2/Twist1 Pathway. Hepatology.

[B76] Wenger C, Kaplan A, Rubaltelli FF (1984). Alkaline phosphatase. Clin Chem.

[B77] Williams JI, Weitman S, Gonzalez CM (2001). Squalamine treatment of human tumors in nu/nu mice enhances platinum-based chemotherapies. Clin Cancer Res.

[B78] Yang ZF, Poon TP (2008). Vascular changes in hepatocellular carcinoma. Anat Rec.

[B80] Zanini C, Giribaldi G, Mandili G (2007). Inhibition of heat shock proteins (HSP) expression by quercetin and differential doxorubicin sensitization in neuroblastoma and Ewing’s sarcoma cell lines. J Neurochem.

[B81] Zhang Y, Ran Y, Yu L (2006). Monoclonal antibody to human esophageal cancer endothelium inhibits angiogenesis and tumor growth. Anticancer Res.

